# The chromogranin A-derived peptides catestatin and vasostatin in dogs with myxomatous mitral valve disease

**DOI:** 10.1186/s13028-020-00541-3

**Published:** 2020-08-05

**Authors:** Katja Höglund, Jens Häggström, Odd Viking Höglund, Mats Stridsberg, Anna Tidholm, Ingrid Ljungvall

**Affiliations:** 1 Department of Anatomy, Physiology and Biochemistry, Faculty of Veterinary Medicine and Animal Science, Swedish University of Agricultural Sciences, 750 07 Uppsala, Sweden; 2 Department of Clinical Sciences, Faculty of Veterinary Medicine and Animal Science, Swedish University of Agricultural Sciences, 750 07 Uppsala, Sweden; 3 Department of Medical Sciences, Faculty of Medicine, Uppsala University, 751 85 Uppsala, Sweden; 4 Anicura Albano Animal Hospital, Rinkebyvägen 21B, 182 36 Danderyd, Sweden

**Keywords:** Canine, Catestatin, Heart, Sympathetic nervous system, Vasostatin

## Abstract

**Background:**

The protein chromogranin A (CgA) is stored and co-released with catecholamines from the stimulated adrenal glands. Increased plasma concentrations of CgA have been shown in people with heart disease. The aim of the study was to investigate whether plasma concentrations of the CgA-derived biologically active peptides catestatin and vasostatin were associated with the severity of myxomatous mitral valve disease (MMVD) in dogs and to assess potential associations between these blood variables and dog characteristics, echocardiographic variables, heart rate (HR), blood pressure (BP) and plasma N-terminal-proBNP (NT-proBNP) concentration. Sixty-seven privately owned dogs with or without MMVD were included. The dogs underwent physical examination, blood pressure measurement, blood sample collection, and echocardiographic examination. Plasma concentrations of catestatin and vasostatin were analyzed using radioimmunoassay.

**Results:**

Catestatin concentration decreased with increasing left atrial and ventricular size (R^2^ ≤ 0.09, P ≤ 0.019), and increased with increasing systolic and diastolic blood pressures (R^2^ ≤ 0.08, P ≤ 0.038). Regression analyses showed no significant associations for vasostatin. No differences in plasma concentrations of catestatin or vasostatin were found between the disease severity groups used in the study.

**Conclusions:**

In the present dog population, the catestatin concentration showed weak negative associations with left atrial and ventricular sizes, both of which are known to increase with increasing severity of MMVD. Furthermore, the catestatin concentration showed weak positive associations with blood pressure.

## Background

Chromogranin A (CgA) is an acidic, soluble secretory protein, belonging to the granin family. The protein has a widespread distribution in neuroendocrine and endocrine tissues as well as in the central and peripheral nervous systems, and is also abundant in the chromaffin cells of the adrenal medulla. Chromogranin A co-exists in secretory granules with catecholamines, with which it is co-released during exocytosis [1–3]. Chromogranin A is also produced in the human myocardium and has been found co-localized with B-type natriuretic peptide (BNP) in the myocardium of people with heart disease [4]. Chromogranin A is a precursor of several biologically active peptides, including catestatin and vasostatin [5], which modulate cardiac hemodynamics during adrenergic stimulation and seem to protect the cardiovascular system against excessive beta-adrenergic stimulation [6].

In contrast to catecholamines, which degrade rapidly in plasma [7], CgA has a high stability [8] and is considered a useful marker of sympathetic nervous activation [9, 10]. In people, plasma CgA has been shown to increase in situations of pronounced sympathetic stimulation such as in critically ill patients [11]. High plasma CgA concentrations have been found in people with congestive heart failure (CHF) due to coronary artery disease, arterial hypertension, dilated cardiomyopathy (DCM), hypertrophic cardiomyopathy and valvular disease [4, 12–14] and plasma CgA concentration has been shown to increase with increasing severity of different human cardiac diseases [12]. Furthermore, studies in people with acute myocardial infarction have shown increased catestatin concentrations in plasma [15–17] as well as in serum [18], while one study showed decreased serum vasostatin-2 concentrations in patients with chronic heart failure due to previous myocardial infarction, compared to in healthy controls [19].

In dogs, myxomatous mitral valve disease (MMVD) is the most common heart disease [20–22]. The disease is characterized by slow progressive degeneration of the mitral valve apparatus, leading to mitral regurgitation (MR). With progressive degeneration of the valve leaflets, the regurgitation increases, leading to volume overload and subsequent dilation of the left atrium and ventricle, and risk of developing CHF [20, 23]. During progression of the disease, neuroendocrine activation takes place [24, 25], and increased plasma norepinephrine concentrations have been found in dogs with DCM and in dogs with advanced MMVD [26, 27].

Plasma concentrations of CgA or derived peptides have not been investigated in dogs with heart disease. A radioimmunoassay (RIA) for measurement of the peptides catestatin and vasostatin, which both have cardiovascular functions [5], has been validated for canine plasma [28]. Hence, the aims of the present study were to investigate whether plasma concentrations of catestatin and vasostatin were associated with severity of MMVD in dogs and to assess potential associations between plasma concentrations of catestatin and vasostatin and dog characteristics, echocardiographic variables, heart rate (HR), blood pressure (BP) and plasma N-terminal-proBNP (NT-proBNP) concentration.

## Methods

### Animals

Client-owned dogs were examined at the cardiology unit at the University Teaching Hospital of the Swedish University of Agricultural Sciences in Uppsala, Sweden according to a pre-specified protocol. To be included, dogs had to either have evidence of MMVD or be free from physical or echocardiographic evidence of cardiac disease. Dogs in need of heart failure therapy were allowed into the study. Dogs with congenital heart disease, other acquired cardiovascular disorders or significant organ-related or systemic diseases were excluded.

### Examinations

Dogs underwent physical examination, blood pressure measurement, blood sample collection, and echocardiographic examination, all performed during the same consultation. Blood pressure was indirectly measured using an automated oscillometric device (Vet HDO monitor, S + B med Vet GmbH, Babenhausen, Germany). Once reliable consecutive readings were obtained, five consecutive blood pressure recordings were performed and the mean was calculated. Blood was collected by jugular venipuncture into EDTA-tubes; plasma was separated by centrifugation within 30 min of collection, transferred to plastic cryotubes and stored at − 80 °C for batched analysis.

Echocardiography, performed by an ultrasonographic unit (iE33, Philips Ultrasound, Bothell, WA, USA), was used to verify the diagnosis of MMVD and to exclude other cardiac diseases. Assessment of mitral valve structures was performed and degree of MR was assessed by color Doppler. The MR was subjectively assessed as the area of regurgitant jet relative to the area of the left atrium [29] with slight modifications [30]. Measurements of the left ventricle (five consecutive cardiac cycles) and left atrial to aortic root (LA/Ao) ratio (three consecutive cardiac cycles) were performed as previously described [31, 32]. The mean value for each variable was used in the statistical analyses. Diagnostic criteria for MMVD included characteristic 2-dimensional valvular lesions of the mitral valve apparatus (thickened and/or prolapsing mitral valve leaflets) and demonstrated MR by color Doppler [29, 33]. Estimation of MMVD severity was based on obtained LA/Ao ratio and MR jet size, and dogs were classified as follows: Healthy (LA/Ao < 1.5 and non to minimal MR jet), mild (LA/Ao ≤ 1.5 and MR jet < 30%), moderate (LA/Ao < 1.8 and MR jet ≤ 50%), and severe (LA/Ao ≥ 1.8 and MR jet > 50%) MMVD [30, 34, 35]. Values for percent increases of end-diastolic left ventricular internal dimension (LVIDd_inc_) and end-systolic left ventricular internal dimension (LVIDs_inc_) were calculated as previously described [30, 36].

### Analyses of catestatin and vasostatin

Analyses of plasma catestatin and vasostatin concentrations were performed by RIAs specific for catestatin and vasostatin [37, 38], validated for use in dogs [28]. The coefficient of variation was 3.6% and 8.8% for catestatin and vasostatin, respectively. The samples had been thawed and refrozen once before the analyses.

### Statistical analyses

Statistical analyses were performed using commercially available software (JMP Pro, version 14.0.0, SAS Institute Inc, Cary, NC, USA). Data are presented as medians and interquartile ranges (IQR). A P-value < 0.05 was considered significant, unless otherwise indicated. The non-parametric Kruskal–Wallis test was used to investigate overall differences between MMVD groups in plasma catestatin and vasostatin concentrations. If a significant difference was detected, pair-wise breed comparisons were performed by Mann–Whitney U-test with Bonferroni adjustment (adjusted P < 0.008). The same statistical methods were used to assess differences between MMVD groups in all basic variables for dog characteristics, clinical, and echocardiographic data, Table 1.

**Table 1:** Dog characteristics, clinical and echocardiographic data in 67 dogs grouped by severity of myxomatous mitral valve disease (MMVD)

Group	Healthy	Mild	Moderate	Severe
Number	20	23	8	16
Sex (female/male)	14/6	15/8	5/3	4/12
Age (years)	4.9 (2.8–6.4)^a^	7.2 (6.2–10)^b^	9.1 (7.0–10)^b^	9.2 (8.7–11)^b^
Body Weight (kg)	8.8 (7.0–10.1)^a^	9.6 (8.0–10.6)^a^	8.9 (7.6–10)^a^	10 (7.7–12)^a^
HR (bpm)	100 (93–111)^a^	100 (92–112)^a^	100 (100–120)^ab^	120 (100–150)^bc^
SBP (mmHg)	134 (119–138)^ab^	139 (132–152)^a^	139 (127–153)^ab^	123 (116–134)^b^
DBP (mmHg)	75 (65–80)^ab^	78 (70–86)^a^	75 (72–80)^ab^	68 (64–75)^b^
LA/Ao	1.2 (1.1–1.2)^a^	1.3 (1.2–1.4)^b^	1.6 (1.5–1.6)^c^	2.1 (2.0–2.6)^d^
LVIDd (cm)	3.0 (2.7–3.4)^a^	3.4 (3.1–3.7)^ab^	3.5 (3.4–3.9)^b^	4.4 (4.1–4.8)^c^
LVIDd_inc_ (%)	4.0 (− 2.4 to 15)^a^	14 (4.5–24)^ab^	24 (16–38)^b^	45 (41–59)^c^
LVIDs (cm)	2.1 (1.8–2.5)^a^	2.3 (2.1–2.7)^a^	2.3 (2.2–2.7)^ab^	2.7 (2.5–3.2)^bc^
LVIDs_inc_ (%)	8.4 (− 0.4 to 30)^a^	21 (12–41)^a^	22 (17–42)^ab^	43 (25–58)^bc^
FS (%)	31 (27–34)^a^	29 (25–34)^a^	35 (31–37)^ab^	37 (31–41)^bc^
Catestatin (nmol/L)	1.2 (1.0–1.3)^a^	1.1 (1.0–1.2)^a^	1.1 (0.9–1.3)^a^	1.1 (0.9–1.2)^a^
Vasostatin (nmol/L)	0.23 (0.15–0.33)^a^	0.18 (0.13–0.28)^a^	0.22 (0.14–0.44)^a^	0.16 (0.11–0.30)^a^
NT-proBNP (pmol/L)	549 (375–800)^a^	473 (386–690)^a^	529 (420–836)^a^	3000 (2129–3000)^b^

Heart rate (HR), systolic blood pressure (SBP), diastolic blood pressure (DBP), echocardiographic data; ratio of left atrium to aortic root (LA/Ao), percentage increase in end-diastolic left ventricular internal dimension, (LVIDd_inc_) and end-systolic left ventricular internal dimension (LVIDs_inc_), fractional shortening (FS), N-terminal-pro B-type natriuretic peptide (NT-proBNP). Values are reported as median and interquartile ranges (IQR). Within each row, values with the same superscript letter did not differ significantly (P > 0.008)

Potential differences between dogs in CHF and dogs without CHF in plasma catestatin and vasostatin concentrations were evaluated by Kruskal–Wallis test, as was potential sex differences in plasma catestatin and vasostatin concentrations.

Univariable regression analyses were performed to evaluate potential associations between age, body weight, systolic (SBP) and diastolic (DBP) blood pressure, HR, NT-proBNP concentration and echocardiographic variables (LA/Ao, LVIDd inc%, LVIDs inc%, fractional shortening (FS)), and plasma catestatin and vasostatin concentrations. To evaluate if potential associations were preserved in dogs not affected by decompensated CHF, the same univariable regression analyses were repeated excluding dogs in decompensated CHF (n = 5). For the univariable regression analyses catestatin, vasostatin, LA/Ao and NT-proBNP concentrations were logarithmically transformed to correct for non-normality.

## Results

Sixty-seven dogs, 38 females and 29 males, with median age 7.7 (IQR 5.9–9.6) years and median body weight 9.6 (IQR 7.7–10.3) kg were included. Summary statistics for the different MMVD severity groups are shown in Table 1. Among the 16 dogs with severe MMVD, five were in decompensated CHF at the time of inclusion, while five dogs had previously been diagnosed with CHF, stabilized by heart failure therapy, and were in compensated CHF at the time of inclusion. In dogs with decompensated CHF at sampling, heart failure therapy was initiated. At the time of sampling, the following number of dogs were treated with furosemide (n = 8), pimobendan (n = 4), ACE-inhibitor (n = 3), digoxin (n = 2) and spironolactone (n = 1).

**Fig. 1:**
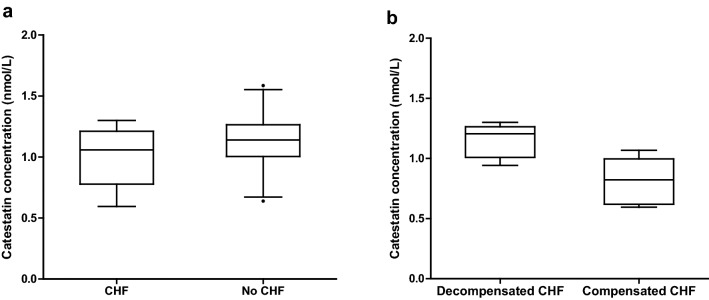
Catestatin concentration in **a** dogs with (n = 10) and without (n = 56) congestive heart failure (CHF) at the time of examination and **b** in dogs in decompensated (n = 5) versus compensated (n = 5) CHF at the time of examination. The top, bottom, and line within each box correspond to the 75th percentile (top quartile), the 25th percentile (bottom quartile), and the 50th percentile (median), respectively. The whiskers extend from the bottom 2.5th percentile to the top 97.5th percentile. Catestatin concentration did not differ between dogs with and without CHF (**a**), P = 0.10. Due to the low number of dogs in each group in **b** statistical comparison could not be performed

Median catestatin concentration in all dogs was 1.13 (IQR 1.00–1.23) nmol/L, with no differences in concentration between MMVD groups. Median vasostatin concentration in all dogs was 0.19 (IQR 0.13–0.30) nmol/L, with no differences in concentration between MMVD groups. Catestatin concentration did not differ between dogs with or without CHF, but was numerically higher in dogs in decompensated CHF compared to in dogs in compensated CHF (statistical comparison not performed due to low number of dogs) (Fig. 1). Catestatin concentration decreased with increasing left atrial size and left ventricular diastolic dimension both in all dogs (n = 67) and when dogs in decompensated CHF were excluded (n = 62); (LA/Ao, R^2^ = 0.08, P = 0.019 in all dogs; R^2^ = 0.12, P = 0.005, decompensated excluded) and (LVIDd inc%, R^2^ = 0.09, P = 0.017 in all dogs; R^2^ = 0.10, P = 0.015, decompensated excluded). Furthermore, catestatin concentration increased with increasing SBP both in all dogs (n = 67) and when dogs in decompensated CHF were excluded (n = 62) (R^2^ = 0.08, P = 0.022 in all dogs; R^2^ = 0.08, P = 0.030, decompensated excluded). Catestatin concentration also increased with increasing DBP in all dogs (R^2^ = 0.06, P = 0.038), but did not reach significance for DBP when dogs in decompensated CHF were excluded (P = 0.052). For vasostatin, univariable regression analyses were non-significant for all included variables and the concentration did not differ between dogs with or without CHF. Catestatin and vasostatin concentrations did not differ between sexes.

## Discussion

In this study of dogs with different severities of MMVD, the plasma catestatin concentration decreased with increasing left atrial and ventricular size, while it increased with increasing systolic and diastolic blood pressure.

The weak negative associations between catestatin concentration and indices of left atrial and ventricular size (R^2^ ≤ 0.09, P ≤ 0.019), were accompanied by a slightly numerically lower catestatin concentration in dogs with CHF compared to dogs without CHF. The small numerical difference was however not significant (Fig. 1).

Several studies have shown increased catestatin concentrations in people with acute myocardial infarction [15–18]. In contrast to the acute course in human myocardial infarction, the common canine cardiac disease MMVD is characterized by a slow but progressive disease development, usually over several years, eventually leading to development of CHF in some dogs [20, 23]. As expected, all indices of left atrial and ventricular size increased gradually with increasing disease severity and the severe MMVD group had significantly higher concentration of NT-proBNP compared to the other groups (Table 1), [39, 40]. The median NT-proBNP concentration of the severe group was approximately six times higher than the other three MMVD severity groups, indicating increased intra-cardiac pressure and volume overload in this group of dogs [41, 42]. Ten of the 16 dogs in the severe group were in CHF and out of these, five were in decompensated CHF. Comparing dogs in decompensated CHF to dogs in compensated CHF, numerically higher catestatin concentration was found in dogs with decompensated CHF (Fig. 1). Due to the small number of dogs (n = 5) in each group, this could not be statistically evaluated, but is in agreement with previous findings of higher plasma norepinephrine concentrations in dogs in decompensated compared to compensated CHF, attributable to MMVD as well as DCM [26, 27]. Hence, the neurohormonal activation in our dogs in decompensated CHF could contribute to their catestatin concentration, but other sources of CgA likely also contribute. Studies suggest that CgA has a widespread distribution in a variety of polypeptide hormone producing human and bovine tissues [3, 43], and certain studies imply that the sympathochromaffin system may be the major source of circulating chromogranin A only in situations of high-intensity sympathetic stimulation, such as in people with sepsis or cardiac arrest [11, 44]. One study in people suggested that at basal to moderate stress levels, norepinephrine and epinephrine concentrations accounted for only 10–15% of the variance in plasma chromogranin A levels [44]. In our dogs, HR was higher in the severe group compared to the healthy and mild groups (Table 1), indicating increased sympathetic activation. However, the median HR in the severe group was only 120 bpm, which is not considered particularly high in dogs in a clinical situation [45]. Thus, potentially the relatively modest sympathetic activation in our dogs could explain the lack of difference between disease severity groups in catestatin and vasostatin concentrations.

Weak positive associations were found between catestatin concentration and systolic as well as diastolic blood pressure (R^2^ ≤ 0.08, P ≤ 0.038). Both SBP and DBP were significantly lower in dogs with severe MMVD compared to the mild group (Table 1), which was an expected finding [34, 46, 47]. In human essential hypertension, CgA has been shown to be elevated while catestatin has been shown to be diminished [48, 49]. This is in contrast to the positive associations between catestatin concentration and BP in this group of dogs. However, the BP values were within or at the upper end of normal limits for all MMVD severity groups [50].

The lower SBP and DBP in dogs with severe MMVD, compared to the mild group, was accompanied by increasing left atrial as well as left ventricular size with increasing disease severity (Table 1). During progression of MMVD, the regurgitant fraction over the mitral valve increases [51], and despite compensatory mechanisms, the left ventricular forward stroke volume might decrease, leading to a decrease in cardiac output and thereby potentially a decrease in SBP [34, 46, 47]. Interestingly, the negative associations between catestatin concentration and indices of left heart size as well as the positive association between catestatin concentration and SBP were maintained even when dogs in decompensated CHF were excluded. This might indicate a potential role of catestatin during progression of the disease. However, the associations found in the present study were weak, and this hypothesis would need to be tested in a larger study population.

The study has limitations. Samples had been thawed and re-frozen once before analysis, which could have affected results. However, because CgA has been shown stable to freezing and thawing [8], this should not have had major effect on the results. While it would have been interesting to measure full-length CgA, only the CgA fragments catestatin and vasostatin have been validated for canine plasma by the RIAs used in our laboratory [28], which precluded that analysis. At the time of sampling, 3 of the 67 dogs were treated with ACE-inhibitors, which might indirectly attenuate sympathetic activity. Excluding these 3 dogs from statistical analyses had no significant effect on the results. Finally, the study population was relatively small with proportionally few dogs in CHF.

## Conclusions

In this population of dogs, the catestatin concentration showed weak negative associations with left atrial and ventricular size, both of which are known to increase with increasing severity of MMVD. Furthermore, the catestatin concentration showed weak positive associations with blood pressure.

## Data Availability

The dataset used and analyzed during the current study are available from the corresponding author on reasonable request.

## Abbreviations

BNP: B-type natriuretic peptide
BP: Blood pressure
CgA: Chromogranin A
CHF: Congestive heart failure
DBP: Diastolic blood pressure
DCM: Dilated cardiomyopathy
FS: Fractional shortening
HR: Heart rate
IQR: Interquartile range
LA/Ao: Ratio of left atrium to aortic root
LVIDd_inc_: Percentage increase in end-diastolic left ventricular internal dimension
LVIDs_inc_: Percentage increase in end-systolic left ventricular internal dimension
MMVD: Myxomatous mitral valve disease
MR: Mitral regurgitation
NT-proBNP: N-terminal-proBNP
RIA: Radioimmunoassay
SBP: Systolic blood pressure
